# Genetic diversity and population structure of critically endangered *Dactylorhiza hatagirea* (D. Don) Soo from North-Western Himalayas and implications for conservation

**DOI:** 10.1038/s41598-022-15742-1

**Published:** 2022-07-09

**Authors:** Shilpa Sharma, Meenu Chhabra, Sunil Kumar Singh, Rajni Parmar, R. K. Kapila

**Affiliations:** Department of Seed Science and Technology, CSKHP Agricultural University, Palampur, H.P 176 062 India

**Keywords:** Biotechnology, Molecular biology, Plant sciences

## Abstract

*Dactylorhiza hatagirea* (D. Don) Soo is medicinally important herb, which is widely used in ayurveda, unani, and folk/traditional medicine system to cure diseases. Due to its immense ethno-botanical properties, the trade of *D. hatagirea* is estimated to be USD 1 billion/year in India. Unfortunately, due to overexploitation of the herb from the wild, has resulted in dwindling of its populations in their natural habitats, which has led to its critically endangered status. Molecular genetic studies are still scarce in *D. hatagirea*, therefore, in current study, genetic diversity and population structure analysis was carried out of 10 populations (48 individuals) collected from three cold desert regions (2527 m–3533 m amsl) of Himachal Pradesh. Mean observed heterozygosity (*H*_*o*_) and expected heterozygosity (*H*_*e*_) was recorded 0.185 and 0.158. The maximum values for *F*_*st*_ (fixation index) and *N*_*m*_ (gene flow) were recorded 0.945 at locus KSSR14 and 1.547 at locus KSSR 4 respectively. Mean genetic differentiation (*F*_*st*_) coefficient was estimated to 0.542. Overall, low levels of genetic diversity was recorded in the populations of *D. hatagirea*, might be due to habitat specificity (alpine meadows ecosystem; humid laden undulating habitat), restricted distribution and high anthropogenic activities. However, two populations viz., Bathad and Rangrik were recorded with high diversity and largest number of private alleles, stipulates that these populations might have high evolutionary significance and response to selection. Dendrogram analysis revealed that the populations of *D. hatagirea* were clustered into four major clusters, which was supported by Bayesian based STRUCTURE predictions. Clustering pattern of majority individuals of different populations revealed consistency with their geographic origin. Outcomes of current study reveals the status of genetic diversity and population structure of endangered *D. hatagirea*, which can be futuristically utilised for appropriate planning of conservation strategies.

## Introduction

Indian Himalayan Region (IHR) is a hotspot for biodiversity due to tremendous variation in climate and altitudinal ranges^1^. This region is the longest bioclimatic gradient which covers approximately 2800 km long area, and is around 220 km–300 km wide along with altitude ranges from 200 to 8000 m^2^. The Indian Himalayan region biodiversity, represents plentiful of angiosperms (8000 species; in which 40% are endemic) and gymnosperms (44 species; in which 16% are endemic)^3^. Abundant flora of the region also acts as “Carbon Sink” which plays an important role in reducing the impact of global warming. Number of plants from IHR has been reported to have immense medicinal and therapeutic values and the Himalayan plants have been valued since Vedic period (Rig Ved 4500-1600BC). It is estimated that, ~ 90% of the medicinally important plant are extracted from wild and 69% of the plant material is collected using destructive harvesting^4^. Increased anthropogenic activities in the Himalayas is considered one of the critical factor responsible for decline in the availability of medicinally important plants and their populations.

*Dactylorhiza hatagirea* (D. Don) Soo belongs to Orchidaceae family, is native to India, Pakistan, Bhutan, Afghanistan, Nepal, and Tibet, is of high value due to its medicinal and ornamental properties. The plant is found at an altitudinal range of 2500–5000 masl in alpine meadows and grassy slopes^5^. It is a monocotyledonous, terrestrial, perennial herb with erect hollow stem and bears purple flowers with green bracts. It is a slow growing, habitat specific species and regenerate poorly due to its requirement of association with mycorrhizae fungi for germination^6^. Bioactive compounds of *D. hatagirea*, which are of medicinal importance are dactyloses A, B, and dactylorhins A to E. Bulbous roots of the plant is used to prepare “Salep” which is utilized to cure wounds, diarrhoea, dysentery, cough, fever, burns and fractures^7^. The plant has high annual demand (5000 tonnes) and price of dried rhizome is ~ 72 US $ per kg, which has resulted in its overexploitation from its natural/wild habitat^4^. Moreover, it has also been observed that *D. hatagirea* in wild shares habitat with highly traded species like *Ophiocordyceps sinensis* and with expanding species like *Polygonum polystachyum*, which is also affecting negatively the number of individuals in a population of *D. hatagirea*^8^. Biodiversity loss is among the most disastrous environmental problem and world is now facing the challenges required to reduce the rate of loss of biodiversity^9^. Moreover, due to the increase in the demand of natural compound in the healthcare system, natural populations of medicinally important plants like *D. hatagirea* are under tremendous pressure due to overexploitation, indiscriminate extraction and unsustainable management.

Understanding of population structure and genetic diversity pattern is important for sustainable utilisation and planning of conservation strategies for critically endangered plant species. Moreover, maintaining genetic variation in a population is one of the most important objective for conserving critically threatened and endangered species. Whole genome sequencing of critically endangered species to create genomic resources are paramount to understand the genome-wide diversity between and within species, which is beneficial for conservation practitioners. Unfortunately, planning of conservation strategies in critically endangered *D. hatagirea* is very challenging due to the non-availability of genetic resources. Till date, only 17 nucleotide sequences^10^ and one transcriptome study has been reported for *D. hatagirea*^11^. Few genetic diversity studies using RAPD and ISSR markers from Ladakh region recorded moderate level of diversity among natural populations^12,13^. Variability among natural populations of *D. hatagirea* from Uttarakhand was also analysed using morphological, biochemical and isoenzymes analysis^14^. However, no population structure and genetic diversity study has been reported from the cold desert regions of Kullu, Kinnaur, Lahaul and Spiti valley of Himachal Pradesh, where *D. hatagirea* is among the most exploited medicinally important plant for domestic usage and is under increasing demand for export^15,16^.

In current study, attempts were made to perform extensive geographic sampling of natural populations of *D. hatagirea*, and samples were collected from 10 locations representing three regions (Kullu, Kinnaur and Lahaul & Spiti) of Himachal Pradesh, India. Fifteen stable and highly polymorphic SSR (Simple Sequence Repeats) markers developed in our previous study were utilised for genetic diversity characterization and population structure analysis of collected natural populations of *D. hatagirea*^10^. SSRs were utilised as they are the most ubiquitous and hypervariable type of repeat elements, which are abundant in the plant genomes. Moreover, SSR markers have added advantages for precise estimate of genetic diversity over other markers because of their co-dominant nature, high reproducibility and transferability^17^. Accordingly, realising the medicinal, ornamental and industrial applications of *D. hatagirea* along with its critically endangered status in the Himalayas, current study was performed with an aim to understand the diversity pattern and population structure of natural populations of *D. hatagirea* for their sustainable management, effective conservation strategy planning and genetic resource management for future breeding programmes.

## Results

### SSR markers and genetic diversity analysis

Diversity analysis of 48 individuals belonging to 10 different sites (Sangla, Chhitkul, Rangrik, Shaloki, Bathad, Tosh, Mane, Sichling, Shego, and Giyu) from three cold desert regions (Kullu, Kinnaur and Lahaul & Spiti) of Himachal Pradesh was performed using six di- and nine tri-nucleotide SSR markers. Initially, for screening of SSR markers, two random individuals from each population were utilised. The number of different alleles (*Na*) varied from minimum 1.1 (KSSR14, KSSR18 and KSSR33) and maximum of 2.0 (KSSR16), number of effective alleles (*Ne*) ranges from minimum 1.047 (KSSR14) and maximum of 1.718 (KSSR16), Shannon's information index (*I*) ranges between 0.05–0.599, observed per locus heterozygosity (*H*_*o*_) ranges from 0.00 (KSSR10) to 0.623 (KSSR16) with mean of 0.185 and expected heterozygosity (*H*_*e*_) varied from 0.032 (KSSR14) to 0.411 (KSSR16) with mean 0.158, respectively. The Polymorphic Information Content (*PIC*) of 15 markers varied from minimum of 0.0939 (KSSR18), 0.1411 (KSSR37) to maximum of 0.5499 (KSSR12), 0.6279 (KSSR16) with an average of 0.3321 (Table 1). Further, genetic differentiation (*F*_*st*_) coefficient and gene flow (*N*_*m*_) analysis at each locus using *F-statistics* revealed significant differences. The maximum values for *F*_*st*_ and *N*_*m*_ were recorded 0.945 at locus KSSR14 and 1.547 at locus KSSR 4, respectively. Mean genetic differentiation (*F*_*st*_) coefficient was estimated to 0.542 for 15 locus ranging from 0.139 (KSSR4) to 0.945 (KSSR14) and mean gene flow recorded was 0.363 ranging from 0.015 (KSSR14) to 1.547 (KSSR4) (Table 1).

**Table 1:** Population genetic parameters of 48 samples representing 10 populations of *Dactylorhiza hatagirea.*

Locus	*Na*	*Ne*	*I*	*Ho*	*He*	*F*	*PIC*	*Fis*	*Fst*	*Nm*
KSSR4	1.800	1.398	0.389	0.355	0.251	− 0.328	0.2392	− 0.412	0.139	1.547
KSSR7	1.400	1.392	0.275	0.271	0.198	− 0.357	0.4319	− 0.371	0.630	0.147
KSSR10	1.300	1.227	0.160	0.000	0.100	1.000	0.2864	1.000	0.701	0.106
KSSR11	1.400	1.101	0.134	0.065	0.074	0.053	0.2416	0.120	0.709	0.103
KSSR12	1.600	1.232	0.254	0.074	0.155	0.448	0.5499	0.521	0.757	0.080
KSSR14	1.100	1.047	0.050	0.040	0.032	− 0.250	0.492	− 0.250	0.945	0.015
KSSR16	2.000	1.718	0.599	0.623	0.411	− 0.483	0.6279	− 0.516	0.409	0.362
KSSR18	1.100	1.100	0.069	0.100	0.050	− 1.000	0.0939	− 1.000	0.474	0.278
KSSR22	1.700	1.338	0.327	0.174	0.207	0.213	0.4174	0.157	0.575	0.185
KSSR30	1.400	1.288	0.240	0.257	0.165	− 0.520	0.2181	− 0.560	0.264	0.695
KSSR32	1.800	1.580	0.474	0.495	0.325	− 0.399	0.3908	− 0.521	0.345	0.475
KSSR33	1.100	1.060	0.056	0.000	0.038	1.000	0.3848	1.000	0.926	0.020
KSSR35	1.500	1.289	0.264	0.260	0.175	− 0.406	0.1823	− 0.486	0.226	0.854
KSSR37	1.200	1.185	0.135	0.000	0.096	1.000	0.1411	1.000	0.348	0.469
KSSR39	1.400	1.152	0.158	0.054	0.097	0.175	0.2839	0.442	0.686	0.114
Mean	1.453	1.274	0.239	0.185	0.158	− 0.126	0.3321	0.008	0.542	0.363

*Na* = Number of Different Alleles, *Ne* = Number of Effective Alleles, *I* = Shannon's Information Index, *Ho* = Observed Heterozygosity, *He* = Expected Heterozygosity, *F* = Fixation Index, *PIC* = Polymorphic Information Content, *Fis* = Inbreeding coefficient among individuals within population, *Fst* = Average genetic differentiation coefficient, *Nm* = Gene Flow.

### Population-wise genetic diversity

Population level genetic diversity parameters were found considerably different across 10 populations of *D. hatagirea* (Table 2). The average of percentage of polymorphic loci (*PPL*) across 10 populations was found moderate (42.67%) which ranged from 20.00% (Chhitkul) to 66.67% (Mane) with only three population revealing *PPL* more than 50% (Mane, Rangrik and Sichling). The number of alleles (*N*_*a*_) per population ranges from 1.200 (Chhitkul) to 1.733 (Mane) with an average of 1.453 and number of effective alleles (*N*_*e*_) varied from 1.139 (Chhitkul) to 1.447 (Tosh) with a mean of 1.274. The observed heterozygosity (*H*_*o*_) varied from maximum of 0.293 (Mane and Sichling) and minimum of 0.080 (Sangla) and expected heterozygosity varied from (*H*_*e*_) 0.235 (Mane) and 0.081 (Chhitkul) with a mean of 0.185 and 0.158 across 10 populations, respectively. Further, the mean value of Shannon's Information Index (*I*) was 0.239 with a range of minimum of 0.119 (Chhitkul) and maximum 0.363 (Mane). The fixation index (*F*) with an average of − 0.126 along with range varied from − 0.084 in Rangrik to 0.242 in Sangla was recorded (Table 2). The maximum polymorphic bands were retrieved for Mane (28.95%), followed by Giyu (26.32%), Rangrik from Spiti valley (21.05%), Bathad from Kullu (15.79%), Sangla from Kinnaur (15.79%), and Sichling from Spiti valley (13.16%) respectively and comparatively low polymorphism was recorded for Chhitkul from Kinnaur (7.89%), Shakoli from Lahaul valley (7.89%) and Tosh from Kullu (5.26%). Total, 20 private alleles were identified in 5 populations viz., Sangla, Rangrik, Bathad, Tosh, and Sichling (Table 2). Presence of the private alleles in these populations stipulates their potential in response to selection and evolutionary significance^18^.

**Table 2:** Genetic variation parameters of the 10 populations of *Dactylorhiza hatagirea* collected from different regions of Himachal Pradesh.

Pop	*Na*	*Ne*	*I*	*Ho*	*He*	*F*	*PPL*	No. Loci with Private Alleles	Name of Loci with Private Alleles
Sangla	1.333	1.187	0.174	0.080	0.115	0.242	33.33%	2	KSSR7
Chhitkul	1.200	1.139	0.119	0.083	0.081	0.022	20.00%	–	**–**
Rangrik	1.600	1.365	0.323	0.240	0.216	− 0.084	60.00%	5	KSSR18
Shakoli	1.333	1.224	0.190	0.167	0.129	− 0.215	33.33%	–	**–**
Bathad	1.400	1.284	0.236	0.147	0.161	0.042	40.00%	10	KSSR11 KSSR14
Tosh	1.533	1.447	0.321	0.317	0.219	− 0.478	46.67%	2	KSSR10
Mane	1.733	1.382	0.363	0.293	0.235	− 0.179	66.67%	–	**–**
Sichling	1.533	1.318	0.276	0.293	0.184	− 0.460	53.33%	–	**–**
Shego	1.333	1.166	0.162	0.083	0.102	0.179	26.67%	1	KSSR33
Giyu	1.533	1.226	0.226	0.143	0.141	0.049	46.67%	–	**–**
Mean	1.453	1.274	0.239	0.185	0.158	− 0.126	42.67%	–	**–**

*Na* = Number of Different Alleles, *Ne* = Number of Effective Alleles, *I* = Shannon's Information Index, *Ho* = Observed Heterozygosity, *He* = Expected Heterozygosity, *F* = Fixation Index, *PIC* = Polymorphic Information Content, *PPL* = Percentage of Polymorphic Loci, *Fis* = Inbreeding coefficient among individuals within population.

### Population genetic differentiation and gene flow

**Figure 1:**
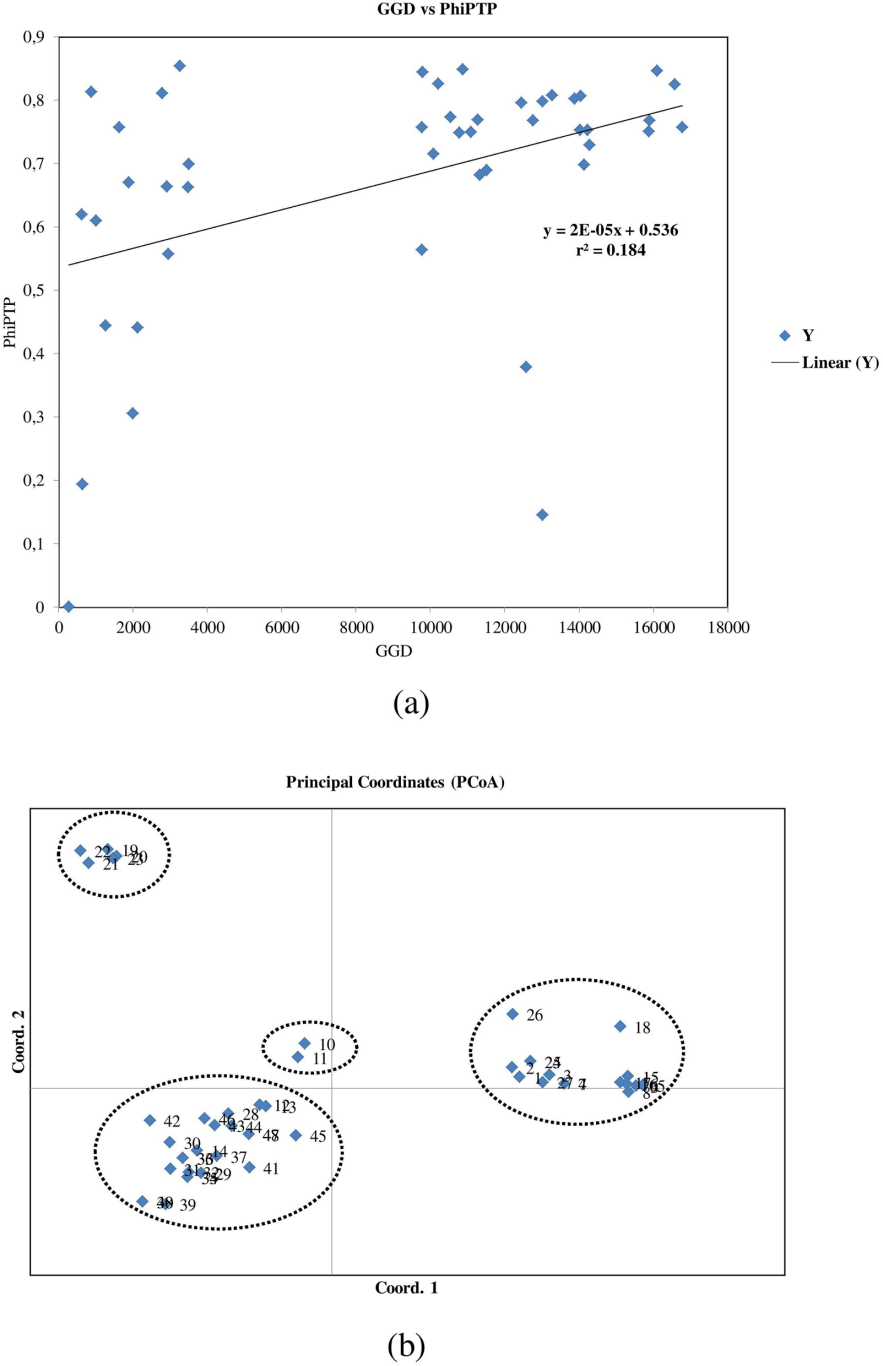
(**a**) Graph representing test of correlation between genetic and geographic distance among 48 individuals of *Dactylorhiza hatagirea*. (**b**) Graph representing Principal Coordinate Analysis (PCoA) of genetic differences among 48 individuals of *Dactylorhiza hatagirea*.

Significant (*P* < 0.001) difference was recorded for the genetic differentiation i.e. *F*_*st*_ between the different pairs of 10 populations, which varied from 0.042 between Sichling and Mane to 0.648 between Bathad and Chhitkul. However, gene flow (*N*_*m*_) values ranges from 0.072 between Chhitkul and Bathad to maximum of 3.071 between Shakoli and Chhitkul (Table 3). Furthermore, pair-wise comparative analysis of Nei’s genetic diversity values revealed variation of 0.019 between Sichling and Mane and 0.891 between Chhitkul and Bathad with majority of the values between populations lies in the range of 0.1–0.7 on the basis of SSR markers (Table 4). Further, Mantel test analysis stipulated weak positive correlation between genetic and geographic distance (*Rxy* = 0.347, *P* = 0.04) indicating isolation-by-distance phenomena in *D. hatagirea* populations (Fig. 1a). Further, AMOVA analysis revealed that 73% of molecular variance was recorded among populations and 27% individual differentiation within populations (Table 5).

**Table 3:** Genetic differentiation coefficient *Fst* (below diagonal) and gene flow *Nm* (above diagonal) between 10 populations of *Dactylorhiza hatagirea*.

Sangla	Chhitkul	Rangrik	Shakoli	Bathad	Tosh	Mane	Sichling	Shego	Giyu	
–	1.595	0.174	0.715	0.091	0.260	0.215	0.176	0.107	0.196	Sangla
0.142	–	0.155	**3.071**	**0.072**	0.317	0.181	0.135	0.086	0.155	Chhitkul
0.357	0.393	–	0.168	0.139	0.149	0.266	0.208	0.281	0.210	Rangrik
0.183	0.117	0.386	–	0.087	0.523	0.194	0.147	0.100	0.152	Shakoli
0.560	**0.648**	0.436	0.584	–	0.123	0.186	0.144	0.096	0.146	Bathad
0.325	0.287	0.462	0.220	0.515	–	0.276	0.214	0.141	0.187	Tosh
0.316	0.368	0.299	0.350	0.376	0.306	–	–	0.632	1.201	Mane
0.349	0.431	0.316	0.384	0.429	0.334	**0.042**	–	0.421	0.799	Sichling
0.487	0.585	0.289	0.514	0.520	0.463	0.172	0.206	–	0.347	Shego
0.300	0.356	0.298	0.345	0.394	0.344	0.107	0.113	0.232	–	Giyu

**Table 4:** *Nei’s* genetic distance represented below diagonal and *Nei’s* genetic identity values represented above diagonal are given for 10 populations of *Dactylorhiza hatagirea*.

Sangla	Chhitkul	Rangrik	Shakoli	Bathad	Tosh	Mane	Sichling	Shego	Giyu	
–	0.961	0.655	0.924	0.470	0.770	0.703	0.711	0.641	0.772	Sangla
0.040	–	0.644	**0.974**	**0.410**	0.829	0.676	0.670	0.633	0.745	Chhitkul
0.424	0.440	–	0.617	0.503	0.455	0.669	0.653	0.775	0.708	Rangrik
0.079	0.027	0.483	–	0.413	0.869	0.649	0.641	0.603	0.698	Shakoli
0.754	**0.891**	0.687	0.883	–	0.451	0.604	0.585	0.505	0.650	Bathad
0.262	0.188	0.788	0.141	0.796	–	0.680	0.671	0.597	0.681	Tosh
0.352	0.392	0.402	0.432	0.504	0.386	–	0.981	0.883	0.932	Mane
0.342	0.401	0.425	0.445	0.536	0.399	**0.019**	–	0.873	0.923	Sichling
0.444	0.457	0.255	0.506	0.683	0.516	0.125	0.136	–	0.866	Shego
0.259	0.294	0.346	0.360	0.431	0.383	0.070	0.080	0.144	–	Giyu

Bold character indicates the highest value, and the lowest values.

**Table 5:** Table representing analysis of molecular variance (AMOVA) among and within populations.

Source	df	SS	MS	Est. Var	%
Among Populations	9	323.808	35.978	6.99	0.73
Within Populations	38	96.921	2.5505	2.55	0.27
Total	47	420.729		9.54	1

Df = Degree of Freedom, SS = Sum of Squares, MS = Mean square, Est. Var. = Estimated Variance.

### Cluster and population genetic structure analysis

**Figure 2:**
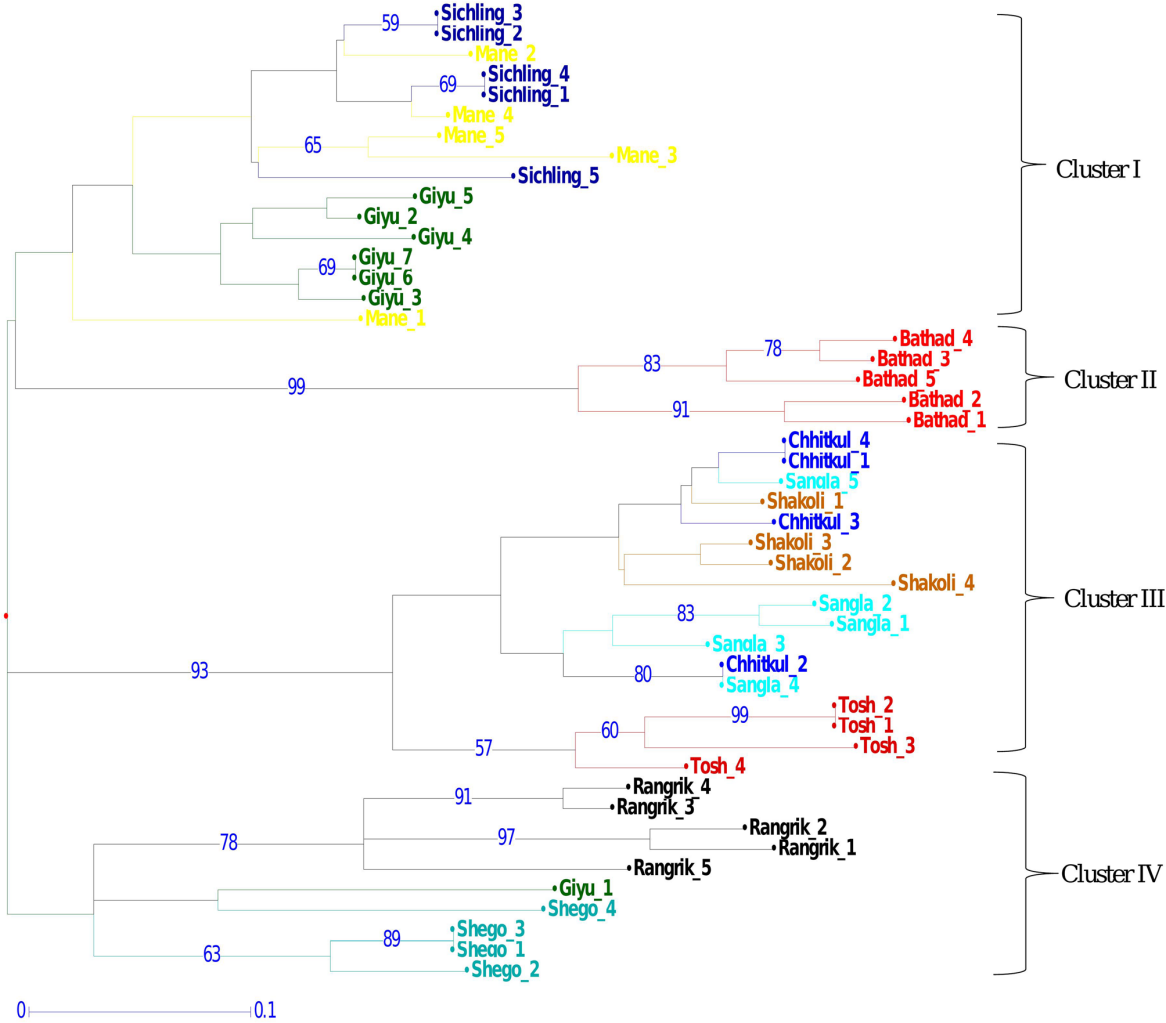
Dendrogram of 10 populations of *Dactylorhiza hatagirea* representing clustering of 48 individuals in four major groups.

**Figure 3:**
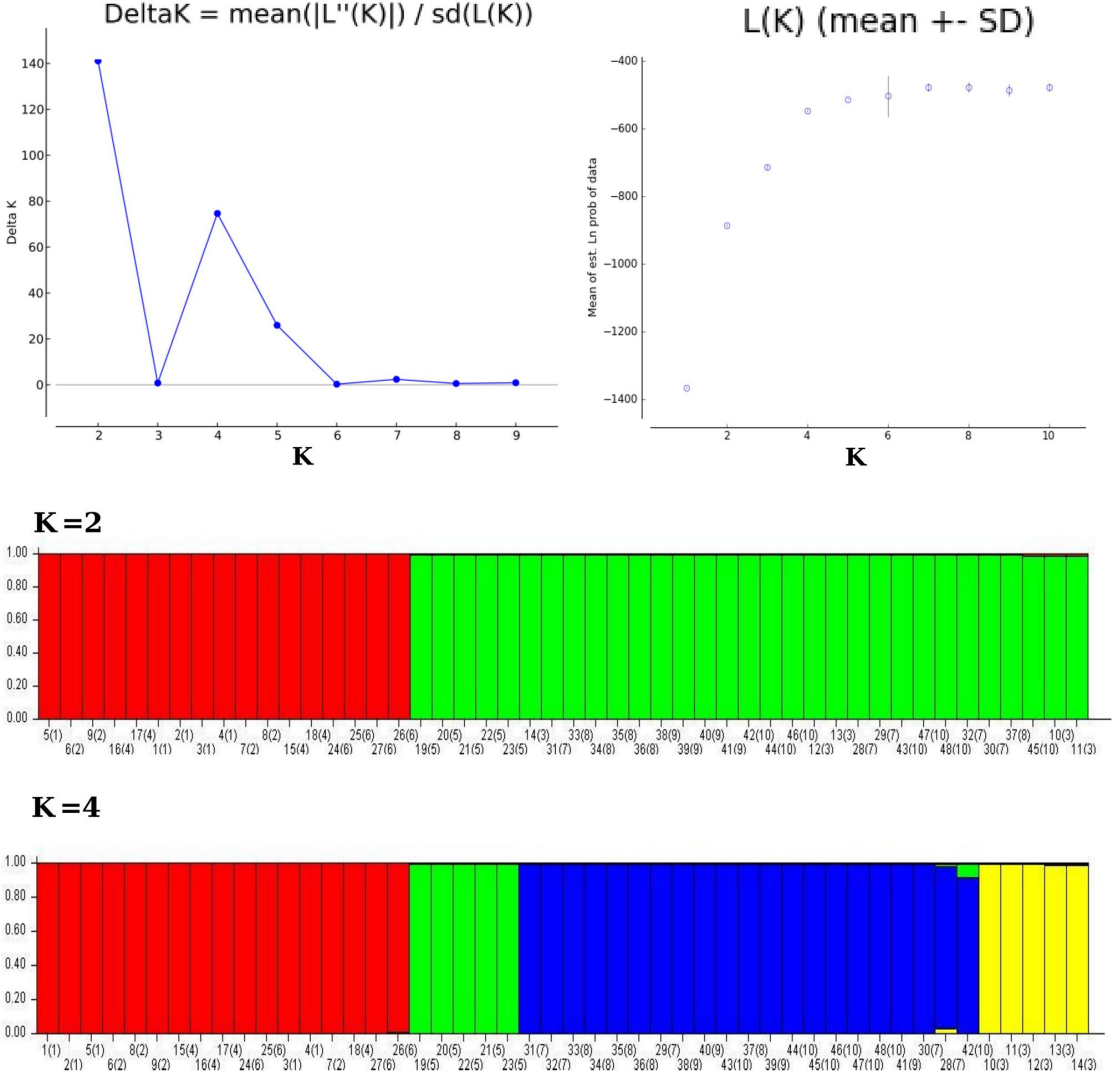
The changes in log-likelihood for different groups D(K), and structure bar plot of 48 individuals (10 populations) of *Dactylorhiza hatagirea* showing pattern of assignment into 4 broad clusters (K = 4). The number in parenthesis indicate populations and outside number are number of individuals.

Dendrogram analysis using Neighbor-Joining based hierarchical clustering revealed that the 10 populations of *D. hatagirea* were clustered into four major clusters *i.e.* cluster I, II, III and IV. Cluster I and IV represented populations from Spiti valley viz*.,* Mane, Sichling, Giyu in cluster I and Rangrik and Giyu in cluster IV. Interestingly, one individual of Giyu (Spiti valley) population was found intermixed with these two populations. Cluster II represented individuals from Bathad population (Kullu) and cluster III represented majority of individuals from Shakoli (Lahaul valley), Tosh (Kullu), Sangla and Chittkul (Kinnaur) (Fig. 2). The Principal co-ordinate analysis (PCoA) analysis also classified the populations into four major groups, which were in line with the clustering analysis (Fig. 1b). Further, the Bayesian based STRUCTURE prediction revealed that maximum likelihood clustering was retrieved when individuals were clustered into two (K = 2) and four groups (K = 4). The K = 2 groups revealed that, *D. hatagirea* populations from Kinnaur (Sangla and Chittkul), Kullu (Tosh), and Lahaul valley (Shakoli) were grouped together in one group and populations from Spiti valley (Mane, Sichling, Shego, Giyu and Rangrik) were grouped together. Further, the maximum likelihood clustering with K = 4 revealed that Bathad population from Kullu region and Rangrik population from Spiti valley belongs to different groups, however, one individual from Mane and Giyu revealed intermixing (Fig. 3).

## Discussion

The drastic changes in the environmental conditions due to climate change and overexploitation by human’s intervention, the risks of extinction of various endangered plants of Himalayan region has increased many times^19^. Orchids are the largest plant families which are commercially traded for various purposes in local, national and international markets. *D. hatagirea* is among the widely utilised and traded Orchid around the world for its potential medicinal properties and health supplements. It has been estimated that approximately 6200 to 31,000 kgs of *D. hatagirea* is harvested annually from the North-Eastern Himalayan region of India^20^. Unfortunately, each kilos of the *D. hatagirea* comprised of around 100 individuals and much of the procurement, harvesting and trade is done illegally and unsustainably, results in its critically endangered status with huge conservation concerns^7^. Understanding the genetic diversity of endangered plant species is of utmost importance, because high genetic diversity is crucial for maintaining the fitness and survival of natural populations, which also ensure the presence of pre-adapted genotypes^21^. Moreover, information related to genetic diversity present within and among populations of endangered plants like *D. hatagirea* is important to establish the conservation strategies^22^.

In current study, genetic diversity of *D. hatagirea* populations from three cold dessert regions of Himachal Pradesh was investigated, because the plant is considered as model to study orchids and their immense pharmacological and therapeutic properties like anticancer, antioxidants and activation of immune system to fight infectious diseases^23^. Despite the medicinal and economical importance of *D. hatagirea* along with its critically endangered status, only few molecular studies using ISSR and RAPD markers have been carried out to understand the genetic diversity and population structure pattern of the *D. hatagirea* populations. However, SSR markers which are widely utilised and recommended for diversity analysis due to their co-dominant and high polymorphic nature has not been used till date to analyse the diversity pattern of *D. hatagirea* populations in the cold desert regions of North-Western Himalayas^24^.

### Genetic diversity analysis based on SSR markers in *D. hatagirea*

In current study, overall low levels of genetic diversity (*H*_*o*_ = 0.185 and *H*_*e*_ = 0.158) was recorded in the populations of *D. hatagirea* which was also reported in earlier studies where genetic diversity of *D. hatagirea* from Ladakh region was estimated using ISSR and RAPD markers^12,13^. The low genetic diversity in few populations of cold desert regions of Kullu, Kinnaur and Lahaul Spiti valley stipulates that the species is under serious threat of extinction in these regions^25^. Moreover, the low genetic diversity recorded might be because of the isolated populations, habitat specificity, restricted distribution and high anthropogenic activities^26^. Moreover, the populations of *D. hatagirea* in alpine meadows ecosystem, prefers humid laden undulating habitat and its population density in alpine meadows usually ambit between 0.70 and 2.43 individuals/m^2^^8,26^. Another possible reason for the low genetic diversity is the small size of the populations and large spatial distance between the populations which hinders the gene flow between the populations, results in self-pollination and inbreeding^24^ In case of *D. hatagirea*, selfing and crossing among related individuals has been reported^12^. All these factors might have resulted into the reduced life span, and health of *D. hatagirea* individuals with exhausted capacity to grow population size^27^.

Current study has reported for the first time the genetic diversity pattern of the 10 populations representing forty-eight individuals from three cold desert regions of Himachal Pradesh. The study reveals the overall variation in the polymorphism level between these populations. Overall, genetic diversity (*I*, *H*_*o*_, *H*_*e*_, and *PIC*) at population level was recorded uniform for majority of populations and was moderated to high in only three populations viz., Mane, Rangrik, Sichling from Spiti valley and one Tosh from Kullu valley. The genetic diversity pattern in these populations indicates low level of human interferences/disturbance in comparison to other populations. Further, individuals from Spiti valley represents the hub for genetic diversity of *D. hatagirea*. However, low levels of genetic diversity in other populations might be due to overexploitation, habitat destruction, poor regeneration due to its specific requirement of growth association with mycorrhiza fungi for germination. Fixation index (*F*) revealed that five populations (Rangrik, Mane, Shakoli, Sichling and Tosh), majority from Lahaul and Spiti valley have negative values, indicating cross pollination or outcrossing and other populations (Sangla, Chhitkul, Bathad, Shego and Giyu) from Kinnaur, Kullu and Spiti valley revealed excess of homozygotes associated, which indicates inbreeding^24^.

### Gene flow, and genetic differentiation

Two important parameters viz., the genetic differentiation coefficient and gene flow plays an important role to understand the genetic structure of the populations and these two parameters are negatively correlated^24,28^. In current study, gene flow between the population of *D. hatagirea* was recorded < 1 (mean *N*_*m*_ = 0.363), which indicates that genetic differentiation is due to genetic drift^29^ and it might be an important factor which is influencing the genetic structure of *D. hatagirea* populations in these regions of Himachal Pradesh. Moreover, Mantel test analysis revealed weak positive correlation between the genetic diversity and geographic distance between populations of *D. hatagirea*. Therefore, the differences in the genetic differentiation might be due to few geographic barriers, resulting in isolating populations in different gene pools of *D. hatagirea*. Further, AMOVA analysis revealed that high molecular variance was recorded among populations and low within populations, which further stipulates habitat fragmentation due to human interventions and anthropogenic activities, leading to low genetic diversity within populations^13^. Moreover, lower genetic variation within populations might be due to vegetative propagation of *D. hatagirea* in the local regions of Western Himalayas.

### Genetic relationship and population structure

**Figure 4:**
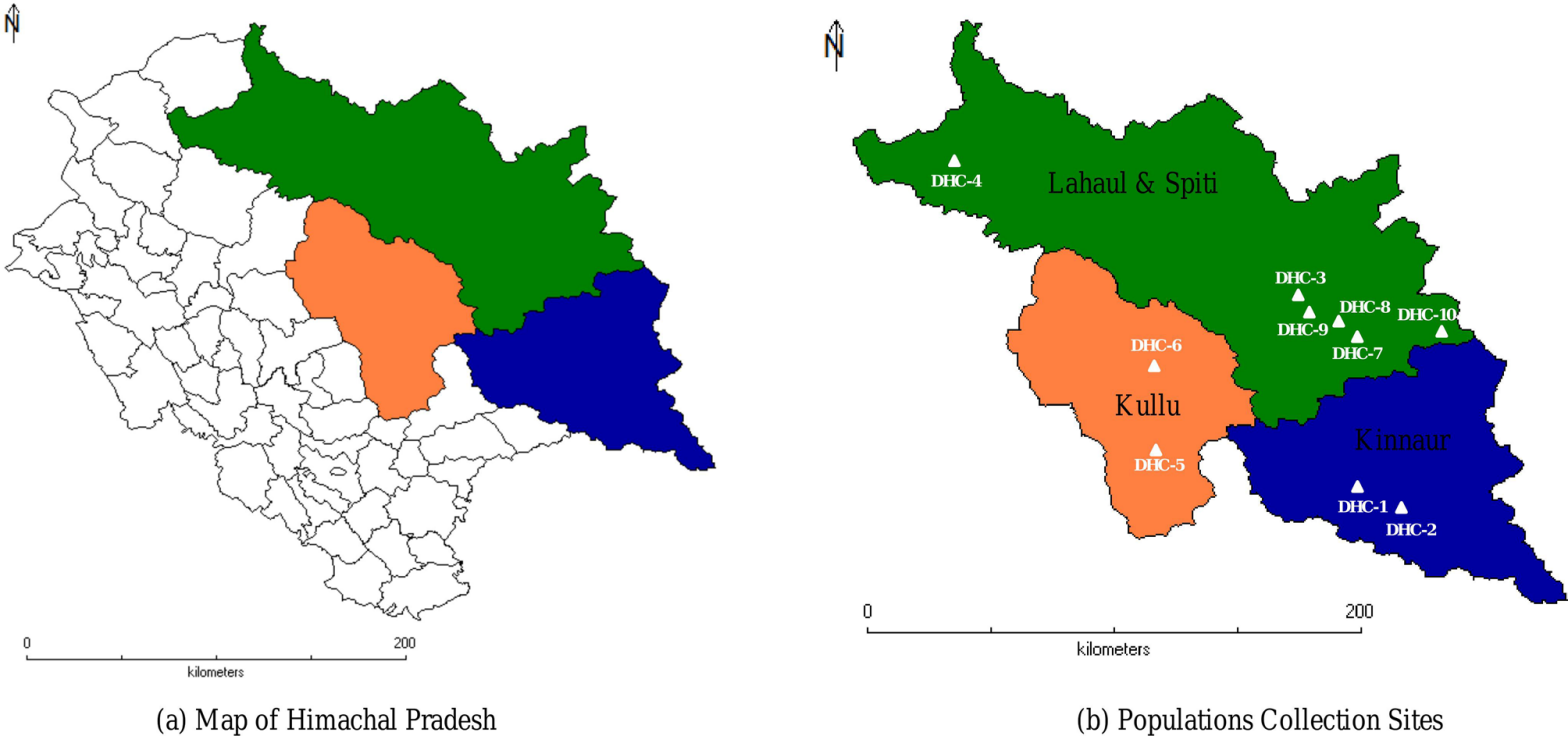
Map representing different sites of collection of 48 individuals of *Dactylorhiza hatagirea.*

The PCoA, UPGMA and STRUCTURE analysis was also performed in the study to predict the genetic diversity and population structure of 48 individuals representing 10 populations of *D. hatagirea*. The distribution pattern of majority individuals of different populations on the basis of these analysis revealed consistency with their geographic origin. The UPGMA analysis grouped the populations into 4 major clusters wherein, cluster I and IV represented population from Spiti valley; cluster II represented population from Kullu and cluster III revealed grouping of populations form Kinnaur, Kullu and Lahaul valley. Despite the long distance between the populations which were represented in cluster III, stipulates low levels of gene flow (mean *N*_*m*_ = 0.363) among them, might be due to collection of *D. hatagirea* from one region and its introduction to the other region. Interestingly, two populations viz., Bathad and Rangrik might be of completely different origin as compare to other populations, also possessed large number of private alleles, and might have role in response to selection and of high evolutionary significance^18^. Structure analysis also clearly suggests that individuals of these populations belong to four major clusters with admixture of few individuals in which populations from Spiti valley seems to be different from rest of the populations. Furthermore, PCoA was also found similar to STRUCTURE results which validate the UPGMA tree. Small admixture retrieved in the structure stipulates the low gene flow (mean *N*_*m*_ = 0.363) among some individuals of 10 populations. Overall, above analysis revealed that it is most likely that majority of the *D. hatagirea* populations in these regions might have been distributed via anthropogenic method and propagated vegetatively, resulted in low population differentiation, gene flow and are genetically similar (Fig. 4).

## Material and methods

### Plant materials

Extensive survey was carried out to identify *D. hatagirea* from wild and leaf samples of forty-eight individuals representing 10 populations (10 sites) from three regions (Kullu, Kinnaur and Lahaul & Spiti) of Himachal Pradesh were collected. Collection of plant material was done without disturbing the natural habitat of the plants and according to the institutional and national guidelines. Moreover, the permission was taken from the Department of Forest, Himachal Pradesh, India and all the samples were collected under their guidance. The formal identification of the plant material was done by ethnobotanist Dr P. K. Sharma, working at Chaudhary Sarwan Kumar Himachal Pradesh Krishi Vishvavidyalaya, Palampur, Himachal Pradesh, India, who has vast experience in identification and research in medicinal and aromatic plants of North-Western Himalayas. The specimen of the plant material has been preserved and deposited to the herbarium of IHBT (Institute of Himalayan Bioresource Technology), Palampur, Himachal Pradesh and field book number has been assigned with specimen voucher no. 27. Tender and healthy leaves of plants which were 10 m apart were sampled before flowering season and immediately frozen in liquid nitrogen container for DNA fixing and then shifted to − 80 °C till further DNA isolation. Leaf samples, a total of 5 individuals from Sangla (Kinnaur; 2616 m amsl), 4 from Chhitkul (Kinnaur; 3393 m amsl), 5 from Rangrik (Spiti valley; 3270 m amsl), 4 from Shaloki (Lahaul Valley; 2991 m amsl), 5 from Bathad (Kullu; 2527 m amsl), 4 from Tosh (Kullu; 2910 m amsl), 5 from Mane (Spiti valley; 3318 m amsl), 5 from Sichling (Spiti valley; 3275 m amsl), 4 from Shego (Spiti valley; 3533 m amsl), and 7 from Giyu (Spiti valley; 3195 m amsl) were collected (Table 6 and Fig. 4).

**Table 6:** Detail of collection of *Dactylorhiza hatagirea* populations from ten sites of Himachal Pradesh along with location, latitude/longitude and altitudinal ranges.

Collections	Plant Numbers	Total Plants Collected	Locations	Districts	State	Latitude/Longitude	Altitude (amsl)
DHC-1	1 to 5	5	Sangla	Kinnaur	Himachal Pradesh, India	31025.114 N 78016.422 E	2616 m
DHC-2	6 to 9	4	Chhitkul	Kinnaur	Himachal Pradesh, India	31021.044 N 78025.284 E	3393 m
DHC-3	10 to 14	5	Rangrik	Spiti Valley	Himachal Pradesh, India	32015.103 N 78002.218 E	3270 m
DHC-4	15 to 18	4	Shakoli	Lahaul Valley	Himachal Pradesh, India	32041.694 N 76040.581 E	2991 m
DHC-5	19 to 23	5	Bathad	Kullu	Himachal Pradesh, India	31051.416 N 77019.024 E	2527 m
DHC-6	24 to 27	4	Tosh	Kullu	Himachal Pradesh, India	31059.375 N 77028.149 E	2910 m
DHC-7	28 to 32	5	Mane	Spiti Valley	Himachal Pradesh, India	32001.609 N 78014.441 E	3318 m
DHC-8	33 to 37	5	Sichling	Spiti Valley	Himachal Pradesh, India	32003.734 N 78013.061 E	3275 m
DHC-9	38 to 41	4	Shego	Spiti Valley	Himachal Pradesh, India	32010.609 N 78006.069 E	3533 m
DHC-10	42 to 48	7	Giyu	Spiti Valley	Himachal Pradesh, India	32004.520 N 78036.737 E	3195 m

### DNA Isolation and PCR amplification

Total genomic DNA extraction was performed using CTAB protocol with little modifications^30^. The quantity and quality of the extracted DNA was estimated using NanoDrop 2000 OD_260_/OD_280_ (Thermo Scientific, Lithuania) and integrity using 1% agarose gel with uncut λ as reference. For PCR amplification, 50 ng of diluted genomic DNA, 0.5 µl of each forward & reverse primers (10 pmol), 0.1 µl of dNTPs (100 µM), 1.5 µl of MgCl_2_ (15 mM), 2.0 µl of 10 × buffer, 0.17 µl (5U/µl) *Taq* DNA polymerase (Genei Labs) was used and final reaction volume was made upto 10.0 µl. PCR reaction was performed using thermal cycler (Applied-biosystems, USA) and amplification cycles were used viz., pre-denaturation at 95 °C for 5 min, 35 cycles of denaturation at 95 °C for 1 min, annealing of primers for 1 min followed by extension at 72 °C for 1.30 min and final extension at 72 °C for 7 min. Amplified PCR products were separated on 6% denaturing PAGE (Poly Acrylamide Gel Electrophoresis) gel and were visualised using silver staining method along with size estimation of amplified product against 50 bp ladder (ThermoFisher).

### SSR data scoring, genetic diversity and population structure prediction

Stable and highly polymorphic fifteen SSR markers (KSSR) were utilised for genotyping of 48 individuals from 10 wild natural populations belonging to three cold desert regions (Kullu, Kinnaur and Lahaul & Spiti) of Himachal Pradesh (Table 7). Presence (1) and absence (0) of bands of a particular size (molecular weight) in the genotyping profiles of all the individuals were scored manually in the binary format. To establish the genetic relationship among collected individuals, each band on the genotyping profile was considered as loci or marker. Scored data for all individuals was utilised for Nei’s gene diversity (*h*), shannon’s information index (*I*), effective number of alleles (*N*_*e*_), percent polymorphic marker loci (*%PPL*), analysis of molecular variance (AMOVA), principal coordinate analysis (*PCoA*), and mantel test of geographic and genetic distance estimation using GenAlEx version 6.5^31^. To understand the genetic relationship among and between populations of *D. hatagirea,* POPGENE version 1.32 was utilised to calculate the gene flow, and *Nei’s* coefficient of genetic differentiation among populations^32^. Further, ARLEQUIN ver 3.5 was used to calculate the *F*_*st*_ and pair-wise *F*_*st*_^33,34^.

**Table 7:** Details of 15 polymorphic microsatellites utilised for diversity analysis.

S. No	Primer	Sequence	Repeat Motif	Ta (°C)
1	KSSR-04	F_CGCGAAGTCAAGATTGAAAA	(TA)6	50
		R_CCCGGCCAGTACTTAACCAG		
2	KSSR-07	F_AAACAAACATGCCCCAGTTA	(TA)6	51
		R_GAGCCGGACATGAGAGTTTC		
3	KSSR-10	F_TCCTCTGCAGTCTTGTTCCA	(TTC)4	55
		R_AAAGCGCATGAGAAAGAACG		
4	KSSR-11	F_TCCTCTGCAGTCTTGTTCCA	(TTC)4	53
		R_GAGAAAGAACGCCAAAGACG		
5	KSSR-12	F_CAGGGGGATAAGTTCTCGAC	(AGA)3	53
		R_AGAAAGAACGCCAAAGACGA		
6	KSSR-14	F_GGTGTTCCTAACTGCCCACT	(TTC)4	54
		R_GAGAAAGAACGCCAAAGACG		
7	KSSR-16	F_CCCGTGGGATTCTTTTTCAT	(GAA)6	53
		R_AGAAAGAACGCCAAAGACGA		
8	KSSR-18	F_CGCGAAGTCAAGATTGAAAA	(TA)6	50
		R_GGGAAATGAACCTTTTGCAC		
9	KSSR-22	F_AAGGTACCACGCTTCGTCAG	(TCT)8	56
		R_GACTGCAGGTAAGGGCTCAG		
10	KSSR-30	F_GCCCGCGAACACTTTATTTA	(TA)8	54
		R_CTCCTCGCGAATGAAATGAT		
11	KSSR-32	F_CGATGGAAGCTGTTCTAACGA	(CAA)3	53
		R_TGGGACTCTCTCTTTATTCTCGTC		
12	KSSR-33	F_CGATGGAAGCTGTTCTAACGA	(AGA)3	55
		R_TGGGACTCTCTCTTTATTCTCGTC		
13	KSSR-35	F_TCAGCGGAGGAGAGGTAGAA	(GAA)6	56
		R_TGGCCACTTGTAGTGAGCTG		
14	KSSR-37	F_CATGCCCCAGTTATCCACTT	(TA)8	53
		R_GAGCCGGACATGAGAGTTTC		
15	KSSR-39	F_TAAACAAACATGCCCCAGTT	(TA)15	50
		R_CTCCTCGCGAATGAAATGAT		

Cluster analysis was performed using *Nei’s* genetic similarity matrix generated in GenAlex from 15 SSR markers using UPGMA (Unweighted Pair Group Method with Arithmetic mean). The data was further utilised to develop a dendrogram using unpaired group mean arithmetic and Jaccard’s similarity coefficients in DARWIN 6.0 software^35^. STRUCTURE analysis based on Bayesian clustering was performed using STRUCTURE version 2.3.4^36^. The population genetic structure (k) was classified by six iterations and value of k was set between “1 to 5” with a burn-in phase 100,000, also Markov Chain Monte Carlo (MCMC) repeats was set 100,000 after burn in and final structure determination was performed using Structure Harvester version 0.6.94^37^.

### Conservation implications

*D. hatagirea* is one of the most important species of Orchidaceae family which is exploited for its bioactive compounds viz., Dactylorhins A-E, and Dactyloses A-B. The species hold immense significance in the clinical and drug discovery research to identify potential bioactive compounds for treatment of diseases associated with respiratory, circulatory and reproductive systems. Unfortunately, due to over-exploitation and unsustainable management, the populations of *D. hatagirea* are dwindling in their natural habitat and loss of genetic diversity within/among these populations further increases the risk of extinction due to factors like inbreeding depression. Moreover, small size of the population also accumulates deleterious genetic variations/mutations due to genetic drift and results in highly vulnerable populations^38,39^. Therefore, increasing the population size is one of the important parameter in conservation of endangered plant species, as effective population size (*N*_*e*_) have huge evolutionary potential and essential to prevent inbreeding depression^40^. The population structure and genetic diversity analysis of *D. hatagirea* revealed low gene flow. Interestingly, populations from two regions viz., Bathad from Kullu and Rangrik from Spiti valley revealed unique and different genetic structure, can be considered as potential populations for conservation strategy planning via introducing them to different regions to induce artificial gene flow^41^. Therefore, conservation of diverse individuals of each population is important to preserve the genetic diversity of *D. hatagirea* in the cold desert regions of Himachal Pradesh as diverse gene pools improves the chances of adaptation for future climatic conditions^42^.
